# Description of a new species of the genus *Eperopeus* Mills, 1967 (Crustacea, Amphipoda, Pardaliscidae) from the Clarion-Clipperton Zone, Central Pacific Ocean

**DOI:** 10.3897/zookeys.1274.139483

**Published:** 2026-03-24

**Authors:** Tammy Horton, Georgina Valls Domedel, Eva C. D. Stewart, Ed A. Hendrycks

**Affiliations:** 1 National Oceanography Centre, Southampton, SO14 3ZH, UK National Oceanography Centre Southampton United Kingdom; 2 School of Ocean and Earth Sciences, University of Southampton, Southampton, SO14 3ZH, UK University of Southampton Southampton United Kingdom; 3 Life Sciences Department, Natural History Museum, London, Cromwell Road, South Kensington, SW7 5BD, UK Canadian Museum of Nature Ottawa Canada; 4 Canadian Museum of Nature, Research Associate, Research and Collections, P.O. Box 3443, Station D, Ottawa, K1P 6P4, Canada Life Sciences Department, Natural History Museum London United Kingdom

**Keywords:** Abyss, amphipods, CCZ, deep sea, identification, key, taxonomy

## Abstract

A new species of the previously monotypic genus *Eperopeus* within the family Pardaliscidae is described from the Clarion-Clipperton Zone in the Pacific Ocean. An updated diagnosis of *Eperopeus* and a key to distinguish the two species is provided.

## Introduction

The genus *Eperopeus* Mills, 1967 was erected for the single species, *E. abyssicola* Mills, 1967, which was described from the western North Atlantic Ocean, near Bermuda, between 2500–4977 m (Mills 1967). *Eperopeus* is unique among the Pardaliscidae in the possession of an entire telson and carposubchelate gnathopods 1–2.

Here we add a new species to the genus *Eperopeus* from the Clarion-Clipperton Zone at depths of 4132–4302 m, extending the geographic distribution of the genus to the Pacific Ocean. The addition of this new species to this rarely collected genus has also allowed us to provide an amended diagnosis, a molecular barcode, and a key to the two species which are very similar morphologically.

## Methods

The material for present study was sampled in the central-east Pacific Ocean, specifically in the easternmost sector of the Clarion-Clipperton Zone (CCZ). The material was collected using either an epibenthic sledge (EBS) or a USNEL Spade Box Core (Ocean Instruments BX-650; BC) during six expeditions to two different exploration contract areas (henceforth, contract areas) in the CCZ: five cruises to the NORI-D (Nauru Ocean Resources Inc.) contract area (C5A in 2020; C5D in 2021; C7A and C7B in 2022; and 8A in 2023) following methods in Glover et al. (2015), and one to the BGR (Bundesanstalt für Geowissenschaften und Rohstoffe) contract area (MANGAN 2016; Rühlemann et al. 2017). For details of gear types and sample processing see the relevant cruise reports and, Jażdżewska et al. (2025).

Specimens were dissected and mounted onto permanent slides using polyvinyl-lactophenol stained with lignin pink. Illustrations were made using Leica M125 and Olympus BX53 microscopes equipped with a camera lucida. Pencil drawings were scanned and inked digitally using Adobe Illustrator and a WACOM digitiser tablet (Coleman 2003, 2009). Some setae are omitted from the illustrations for clarity. Appendages of the right side are dissected and illustrated, unless otherwise stated.

The following abbreviations were used: **A1, A2** = antenna 1, 2; **G1, G2** = gnathopod 1, 2; **LL** = lower lip; **Md** = mandible; **Mx1, Mx2** = maxilla 1, 2; **Mxp** = maxilliped; **P3–P7** = pereopod 3–7; **T** = telson; **U1–U3** = uropod 1–3; **UL** = upper lip; **l** = left; **r** = right.

Type material is deposited in the Natural History Museum, London (**NHMUK**). Additional material is kept in the Deutsches Zentrum für Marine Biodiversitätsforschung (**DZMB**) in Wilhelmshaven, the Discovery Collections at the National Oceanography Centre, Southampton (**DISCOLL**), and the Canadian Museum of Nature, Ottawa (**CMNC**).

### DNA extraction, amplification, and sequencing

Specimens collected from the MANGAN cruises were extracted and sequenced as described in Jażdżewska et al. (2025). Specimens collected from the NORI-D contract area were processed as follows.

DNA was extracted from a pair of pleopods using QuickExtract^TM^ DNA extraction solution (Lucigen), following manufacturer guidelines, and adapted for a digestion time of 45 minutes. Regions of two mitochondrial [16S rRNA (16S) and cytochrome c-oxidase subunit I (COI)] and two nuclear [28S rRNA (28S), and early-stage histone 3 (H3)] genetic markers were amplified with published primer sets (Astrin and Stüben 2008; Corrigan et al. 2014; Lörz et al. 2018). The PCR mix for each reaction contained 10.5 µl of Red Taq DNA Polymerase 1.1 × MasterMix (VWR), 0.5 µl of each primer (10 µM), and 1 µl of DNA template. Primers and PCR conditions are detailed in Table 1.

**Table 1.** Primers and PCR programs used for DNA amplification.

**Gene**	**Primer**		**Sequence (5’ – 3’)**	**PCR program**	**Reference**
COI	LCO1490-JJ	Forward	CHACWAAYCATAAAGATATYGG	1 × (2 min at 94 °C), 5 × (30 s at 94 °C, 90 s at 45 °C, 60 s at 72 °C), 35 × (30 s at 94 °C, 90 s at 51 °C, 60 s at 72 °C), 1 × (5 min at 74 °C)	Astrin and Stüben 2008
	HCO2198-JJ	Reverse	AWACTTCVGGRTGVCCAAARAATCA		Astrin and Stüben 2008
16S	16SFt_amp	Forward	GCRGTATIYTRACYGTGCTAAGG	1 × (2 min at 95 °C), 35 × (30 s at 95 °C, 30 s at 50 °C, 45 s at 72 °C), 1 × (5 min at 72 °C)	Lörz et al. 2018
	16SRt_amp	Reverse	CTGGCTTAAACCGRTYTGAACTC		Lörz et al. 2018
28S	28Sftw	Forward	AGGCGGAATGTTGCGT	1 × (2 min at 95 °C), 35 × (40 s at 94 °C, 40 s at 50 °C, 40 s at 72 °C), 1 × (10 min at 72 °C)	Corrigan et al. 2014
	28Srtw	Reverse	CTGAGCGGTTTCACGGTC		Corrigan et al. 2014
H3	HisH3f	Forward	AAATAGCYCGTACYAAGCAGAC	1 × (2 min at 95 °C), 35 × (40 s at 94 °C, 40 s at 45 °C, 40 s at 72 °C), 1 × (10 min at 72 °C)	Corrigan et al. 2014
	HisH3r	Reverse	ATTGAATRTCYTTGGGCATGAT		Corrigan et al. 2014

The primers used for sequencing were the same as those for amplifications. PCR products were purified using a Millipore Multiscreen 96-well PCR Purification System and sequenced using an ABI 3730XL DNA Analyzer (Applied Biosystems) at The Natural History Museum Sequencing Facilities. For each gene fragment contigs were assembled by aligning both forward and reverse sequences, chromatograms were visually inspected, and ambiguous base calls were corrected manually, using Geneious 7.0.6 (Kearse et al. 2012).

All sequences were deposited in GenBank with the accession numbers provided in the material sections. For specimens collected during MANGAN cruises, voucher information, taxonomic classifications and sequences are deposited in the data set “DS-AMPHICCZ” in the Barcode of Life Data System (BOLD) ((https://doi.org/10.5883/DS-AMPHICCZ) (http://www.boldsystems.org) (Ratnasingham and Hebert 2007).

## Results

### Systematics

**Order AMPHIPODA Latreille, 1816**

**Suborder AMPHILOCHIDEA Boeck, 1871**

**Superfamily DEXAMINOIDEA Leach, 1814**

**Family PARDALISCIDAE Boeck, 1871**

#### Genus Eperopeus

Taxon classificationAnimaliaAmphipodaPardaliscidae

Mills, 1967

7C968020-FEFE-5B38-81B0-87FEC96DBA2D

Eperopeus Mills, 1967: 351.―Karaman 1974: 10; Barnard and Karaman 1991: 576; Biswas et al. 2009: 24 (in key); Paz-Rios and Pech 2022: 2.

##### Type species.

*Eperopeus abyssicola* Mills, 1967 (original designation).

##### Diagnosis.

Head lacking rostrum. Antennae short, equal in length or antenna 2 slightly longer than antenna 1. Coxae all very shallow, subovate or subquadrate. Gnathopods 1 and 2 carposubchelate; carpus elongate, broad medially; propodus short, not expanded; palm lacking. Mandible, incisor toothed; palp article 2 expanded. Maxilla 1, palp article 2 expanded distally. Maxilliped, inner plate small, narrow; palp longer than inner edge of outer plate. Pereopods 5 and 6 with long slender dactyls. No dorsal processes on urosome in either sex. Uropods 1 and 2 rami without setae. Uropod 3 short, outer ramus two-articulate. Telson entire. (Amended after Mills 1967).

##### Remarks.

The genus *Eperopeus* was erected for the single species, *E. abyssicola*, which was described from the western North Atlantic Ocean, near Bermuda, between 2500–4977 m (Mills 1967). The genus *Eperopeus* is unique among the Pardaliscidae in possession of an entire telson and carposubchelate gnathopods 1–2.

The recently described genus *Paraeperopeus* Paz-Rios & Pech, 2022 has similar gnathopods, with the carpus longer than the propodus and broadened medially, but differs in having a long, distinct rostrum (lacking in *Eperopeus*), setose ventral margins of coxae 1–4, and a slightly emarginate telson.

*Eperopeus* also shares the characteristic gnathopods with *Necochea* Barnard, 1962, *Pardaliscoides* Stebbing, 1888, and *Princaxelia* Dahl, 1959, from which it is distinguished by numerous morphological characters in addition to the entire telson. The only other genus with an entire telson is *Parpano* Barnard, 1964 (Caribbean Sea), from which *Eperopeus* can be distinguished by, among many other characters, the broadened carpus of gnathopods 1–2 (carpus is strongly shortened in *Parpano*), very shallow coxae (deep in *Parpano*), and stout mandibular palp (slender in *Parpano*).

##### Species.

*Eperopeus abyssicola* Mills, 1967; *Eperopeus vermiculatus* sp. nov.

#### Eperopeus vermiculatus sp. nov.

Taxon classificationAnimaliaAmphipodaPardaliscidae

0C97B462-9585-5C38-9689-1219F56C5CBD

https://zoobank.org/B7BE05F0-F4CE-4E42-8561-66A55A22C937

Figs 1, 2, 3, 4, 5

##### Type material.

***Holotype***: Pacific • female (with setose oostegites), 3.6 mm (13 slides); Clarion-Clipperton Zone; 10.3773°N, 117.1558°W; depth 4302 m; 14/05/2021; NORI-D contract area, RV *Maersk Launcher*, Cruise C5D, Station STM_168, Box Core BC_395; NHMUK 2025.28 (Specimen 7249_TH_AMP_1), 16S (PV075086). ***Paratypes***: Pacific • female (without oostegites), 2.9 mm; Clarion-Clipperton Zone; 10.8397°N, 116.1504°W; depth 4132 m; 25/05/2021; NORI-D contract area, RV *Maersk Launcher*, Cruise C5D, Station SPR_160, Box Core BC_412; NHMUK 2025.29 (Specimen 8233_TH_AMP_1) • female (with setose oostegites), 4.1 mm; Clarion-Clipperton Zone; 10.3315°N, 117.1872°W; depth 4284 m; 28/05/2021; NORI-D contract area, RV *Maersk Launcher*, Cruise 5D, Station STM_195, Box Core BC_415; NHMUK 2025.30 (Specimen 8571_TH_AMP2), 16S (PV075086), 28S (PV075089) • female (without oostegites), 2.9 mm; Clarion-Clipperton Zone; 10.3559°N, 117.1688°W; depth 4277 m; 11/11/2020; NORI-D contract area, RV *Maersk Launcher*, Cruise 5A, Station STM_010, Box Core BC_355; NHMUK 2025.31 (specimen 8803_TH_AMP1) • Male (with expanded callynophore), 4.3 mm; Clarion-Clipperton Zone; 10.3336°N, 117.186°W; depth 4279 m; 02/12/2022; NORI-D contract area, MV *Island Pride*, Cruise 7B, Station TF_007_01, Box Core BC_478; NHMUK 2025.32 (specimen 10333_TH_AMP_1), COI (PV077102), 16S (PV075087), 28S (PV075090), H3 (PV078005).

##### Other material.

Pacific • female, 4.0 mm; Clarion-Clipperton Zone; 10.3773°N, 117.1558°W; depth 4302 m; 14/05/2021; NORI-D contract area, RV *Maersk Launcher*, Cruise C5D, Station STM_168, Box Core BC_395; DISCOLL-TMC-AMP-7251-a (specimen 7251_TH_AMP1) • female (no obvious oostegites), 2.3 mm; Clarion-Clipperton Zone; 10.3553°N, 117.169°W; depth 4274; 09/09/2022; NORI-D contract area, MV *Island Pride*, Cruise 7A, STM_010, Box Core BC_337; DISCOLL-TMC-AMP-9393-a (specimen 9393_TH_AMP_1) • female, 3.1 mm; Clarion-Clipperton Zone; 10.3281°N, 117.1817°W; depth 4286 m; 24/11/2023; NORI-D contract area, MV *Coco*, Cruise 8A, Station IM013_BCR2, Box Core BC487; DISCOLL-TMC-AMP-12535-a (specimen 12535_TH_AMP_1), COI (PV077103), 16S (PV075088) • female (with oostegites), 4.5 mm; Clarion-Clipperton Zone; 10.3541°N, 117.2424°W; depth 4290 m; 13/12/2023; NORI-D contract area, MV *Coco*, Cruise 8A, Station IM003_BC, Box Core BC501; CMNC 2025-0001 (specimen 12548_TH_AMP_3) • male; not measured, Clarion-Clipperton Zone; 11.892°N, 117.489°W; depth 4193–4256 m; 10/05/2016; BGR contract area, RV *Kilo Moana*, Cruise MANGAN 16, Station SO 262-96, Epibenthic Sledge; DSB_3704, COI (PQ734521).

##### Type locality.

Abyssal Pacific Ocean, Clarion-Clipperton Zone, 10.3773°N, 117.1558°W; depth 4302 m.

##### Diagnosis.

Gnathopod dactyls inner margin lacking teeth; gnathopod 2 carpus narrower than that of gnathopod 1; pereopods 3–4 dactyls short, length 0.8–0.9 × respective propodus; pereopods 5–6 dactyls length 1.2 × propodus; mandibular palp article 1 elongated, 0.9 × length of article 2; maxilla 1 palp article 2 widened; uropod 1 rami length 0.8 × peduncle; telson longer than wide, bluntly rounded distally, length 1.1 × width.

##### Description.

Based on holotype female with setose oostegites, 3.6 mm, NHMUK 2025.28.

**Figure 1.**
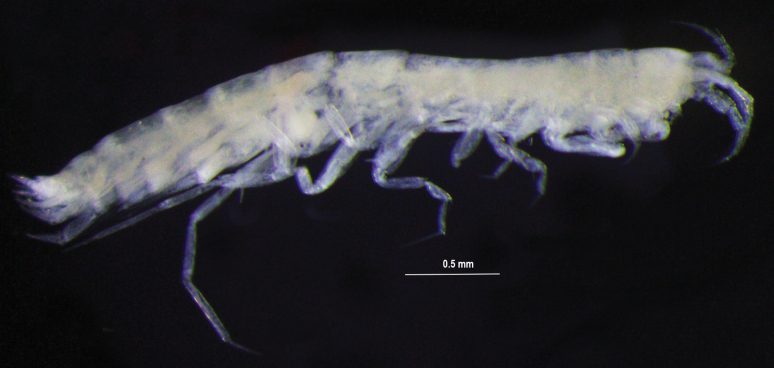
*Eperopeus vermiculatus* sp. nov., habitus of the female holotype, 3.6 mm; NHMUK 2025.28. Photograph of preserved specimen (T. Horton). Scale bar: 0.5 mm.

**Figure 2.**
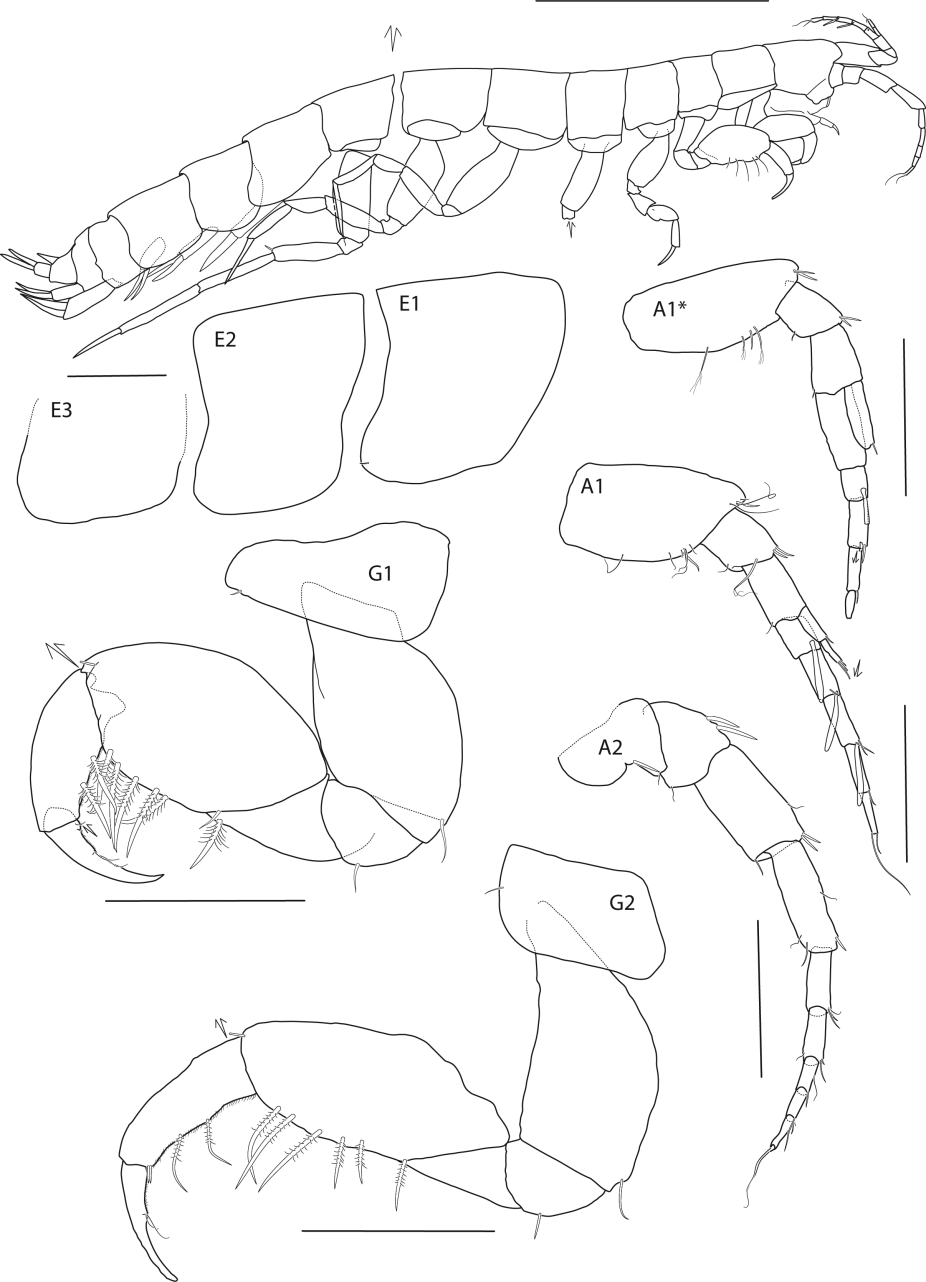
*Eperopeus vermiculatus* sp. nov., female holotype (with setose oostegites), 3.6 mm; NHMUK 2025.28. Scale bars: 1.0 mm (habitus); 0.2 mm (Epimeres, A1, A2, G1, G2); A1* = male A1.

***Body*** (Figs 1, 2): vermiform, pereonites and pleonites subequal or longer than deep. ***Pleonite 1*, *2*** (Fig. 2) smooth, lacking dorsal teeth. ***Urosomite 1*** (Fig. 2) smooth, lacking dorsal teeth. ***Epimeron 1*** (Fig. 2) with a rounded posterodistal corner and a small seta. ***Epimeron 2*, *3*** (Fig. 2) subquadrate, anterodistal corner broadly rounded, distal margin convex, posterodistal corner not produced, posterior margin straight. ***Coxae 1*–*7*** (Fig. 2) reduced and shallow, much shorter than corresponding pereonites.

***Head*** (Fig. 2): shorter in length than pereonites 1–2 combined; rostrum not obvious. ***Lateral cephalic lobe*** broadly triangular. ***Eye*** not apparent in preserved specimens. ***Antenna 1*** very short, length 0.17 × body, geniculate; peduncular article 1 length 1.8 × width, angled on lateral margin so inner margin is longer; peduncular articles 2–3 short, length less than peduncle 1; flagellum five-articulate, first article of flagellum not callynophorate, each article with a large aesthetasc; accessory flagellum short, two-articulate, first article slightly broader. ***Antenna 2*** slightly longer than antenna 1, gland cone small; peduncular articles 4–5 subequal, very weakly spinose and setose; flagellum five-articulate.

**Figure 3.**
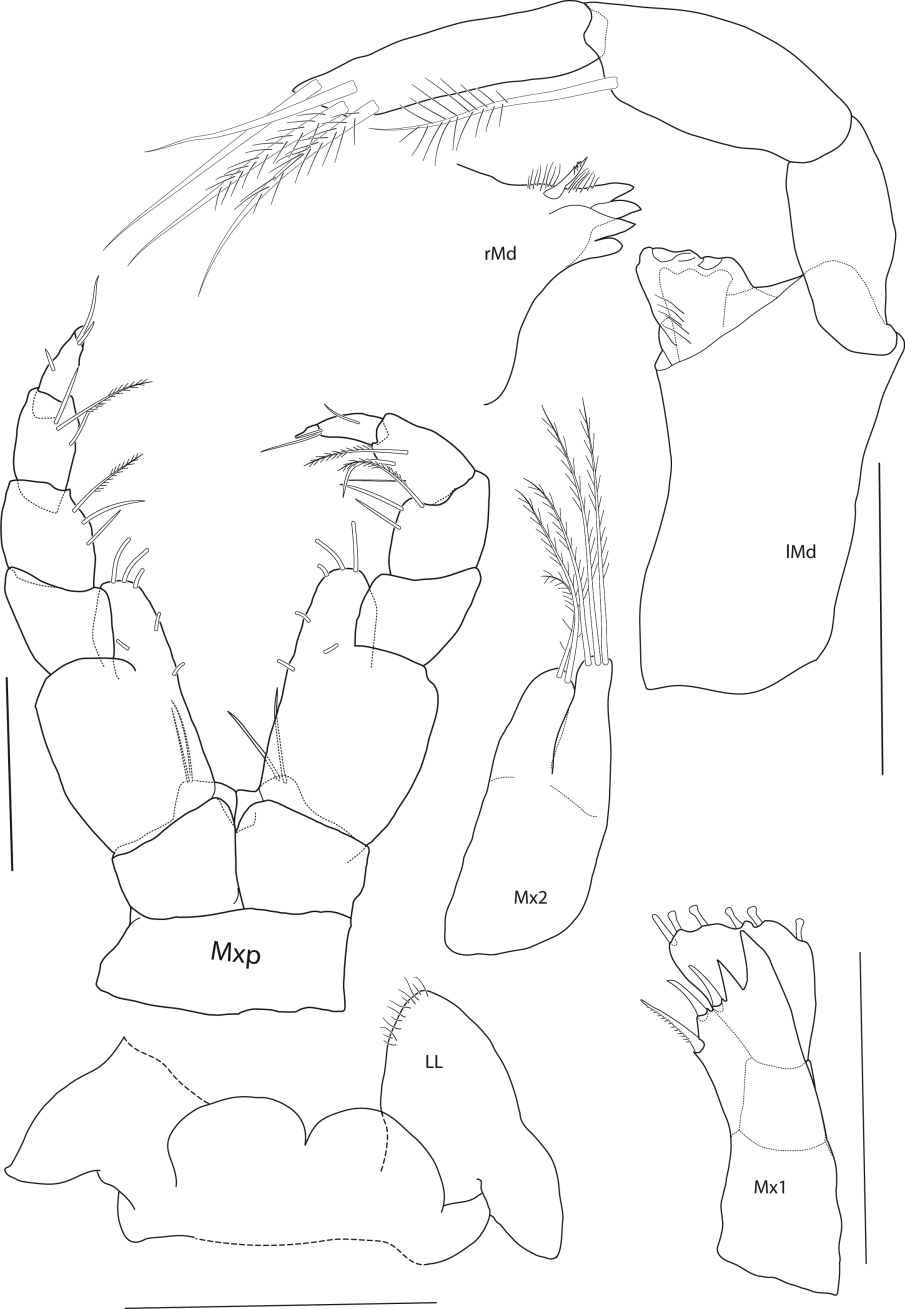
*Eperopeus vermiculatus* sp. nov., female holotype (with setose oostegites), 3.6 mm; NHMUK 2025.28. Scale bars: 0.1 mm (Mxp, Mx2, Mx1, LL, Md, lMd).

***Mouthparts*** (Figs 2, 3): ***Epistome*** (Fig. 2) level with and scarcely differentiated from upper lip. ***Mandible*** (Fig. 3) incisor weakly dentate; left lacinia mobilis three-dentate; accessory spine row with four setae; molar absent; palp strong, article 1 elongate, length 2.36 × width and 0.9 × length of article 2, article 2 slightly shorter than article 3, not expanded, with one distal seta, article 3 cylindrical, subacute distally, with four strong setae; right mandible incisor four-dentate, teeth large, accessory spine row with one spine and fine setae. ***Lower lip*** (Fig. 3) outer lobe broad, mandibular lobe narrow. ***Maxilla 1*** (Fig. 3) inner plate missing; outer plate with four strong teeth; palp two-articulate, article 2 strongly widened distally, with six nodular setae. ***Maxilla 2*** (Fig. 3) slightly reduced, inner plate slightly shorter and broader than outer, tapering distally, both with long pappose apical setae. ***Maxilliped*** (Fig. 3) inner plate reduced, distal margin with two long setae; outer plate subovate, reaching distomedial end of palp article 1, with two apical and three medial setae, medial margin straight; palp short, weakly setose medially, articles 1–2 slightly expanded, article 3 narrow, article 4 long, length 0.8 × article 3.

**Figure 4.**
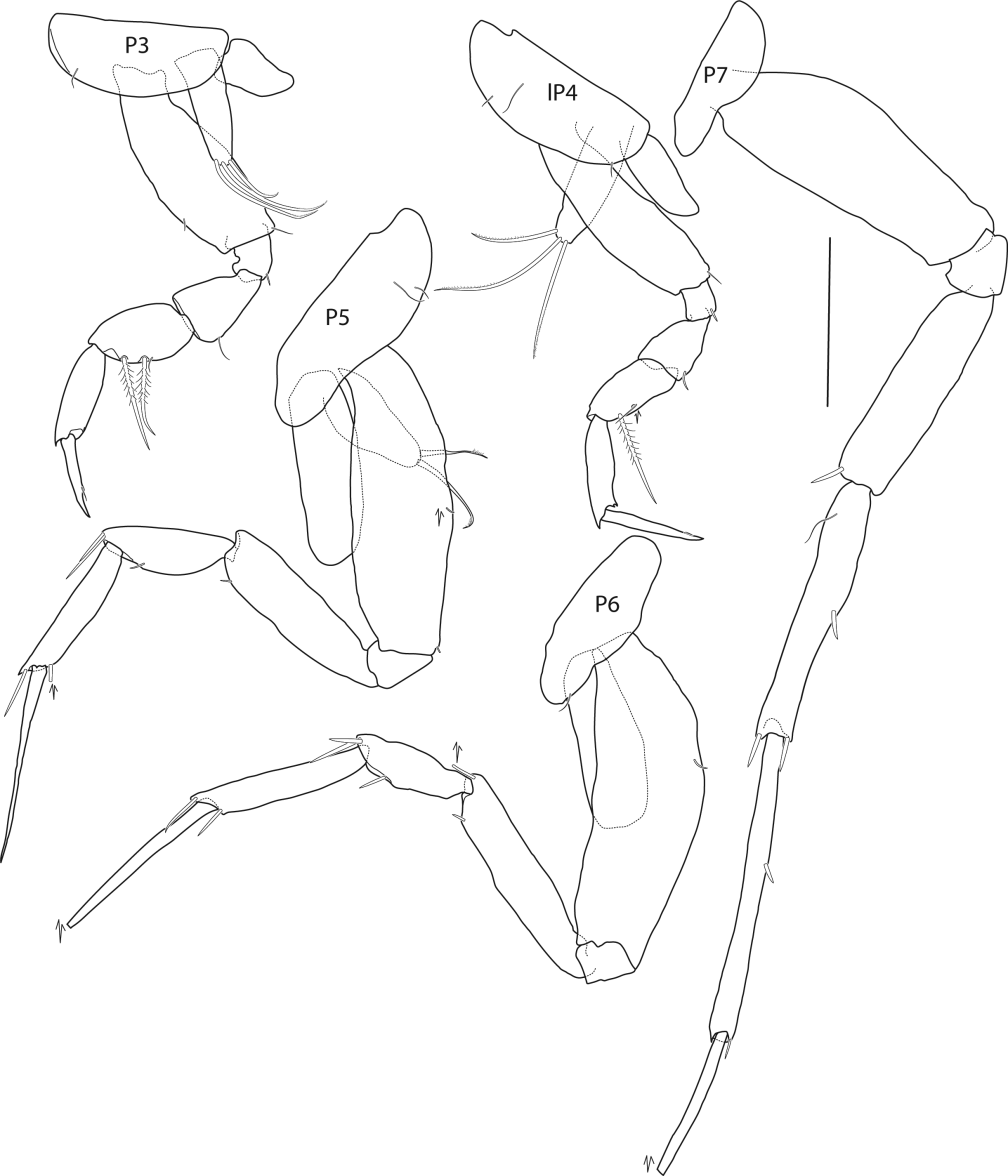
*Eperopeus vermiculatus* sp. nov., female holotype (with setose oostegites), 3.6 mm; NHMUK 2025.28. Scale bar: 0.2 mm (P3–P7).

***Pereon*** (Figs 2, 4): ***Gnathopod 1*** (Fig. 2) carposubchelate; coxa narrow, length 2.5 × width, anterodorsal corner rounded, posterior margin nearly straight, distal margin straight; basis short, strongly expanded, length 1.3 × width; ischium shorter than merus; carpus broad, length 1.6 × width, posterior margin with five strong pectinate setae; propodus narrow and curved, length 0.64 × carpus, posterior margin with two strong pectinate setae, lacking palm; dactylus narrow, inner margin lacking teeth. ***Gnathopod 2*** (Fig. 2) carposubchelate; coxa subrectangular, length 1.7 × width, anterodorsal corner rounded, posterior margin convex, distal margin straight; basis short, expanded, length 2.1 × width: ischium shorter than merus; remaining articles as in gnathopod 1 except carpus narrower, length 2.4 × width. ***Pereopod 3*** (Fig. 4) coxa crescent-shaped, shallow, length 2.3 × width, ventral margin convex; merus widening distally, triangular, slightly shorter than carpus; carpus dilated, ovate, posterior margin with two long pectinate setae; propodus subequal in length to carpus, anterodistal corner acute; dactylus straight, length 0.8 × propodus. ***Pereopod 4*** (Fig. 4) similar to pereopod 3 except coxa longer; merus slightly narrower; carpus narrow and dactylus longer, length 0.9 × propodus. ***Pereopod 5*** (Fig. 4) coxa narrow, length twice width, anterodistal and posterodistal margins rounded; basis narrow, length 3.2 × width, anterior and posterior margins slightly convex; merus length 0.7 × carpus-propodus; carpus, posterodistal corner with two spines; propodus narrow, longer than carpus, posterodistal corner acute with spine; dactylus straight, length 1.2 × propodus. ***Pereopod 6*** (Fig. 4) coxa narrowly ovate, length 2.9 × width; basis narrow, length 3 × width, anterior margin strongly convex, posterior margin slightly concave; rest of pereopod articles as in pereopod 5. ***Pereopod 7*** (Fig. 4) long, length 1.3 × pereopod 5–6; coxa small, narrowing posteriorly, margins round; basis narrow, length 2.8 × width, anterior margin convex proximally, posterior margin straight; merus slightly shorter than carpus, with one posterodistal spine; carpus long, anterior margin with two spines, posterodistal corner with one spine; propodus long, length 1.2 × carpus, with two spines; dactylus partially missing.

**Figure 5.**
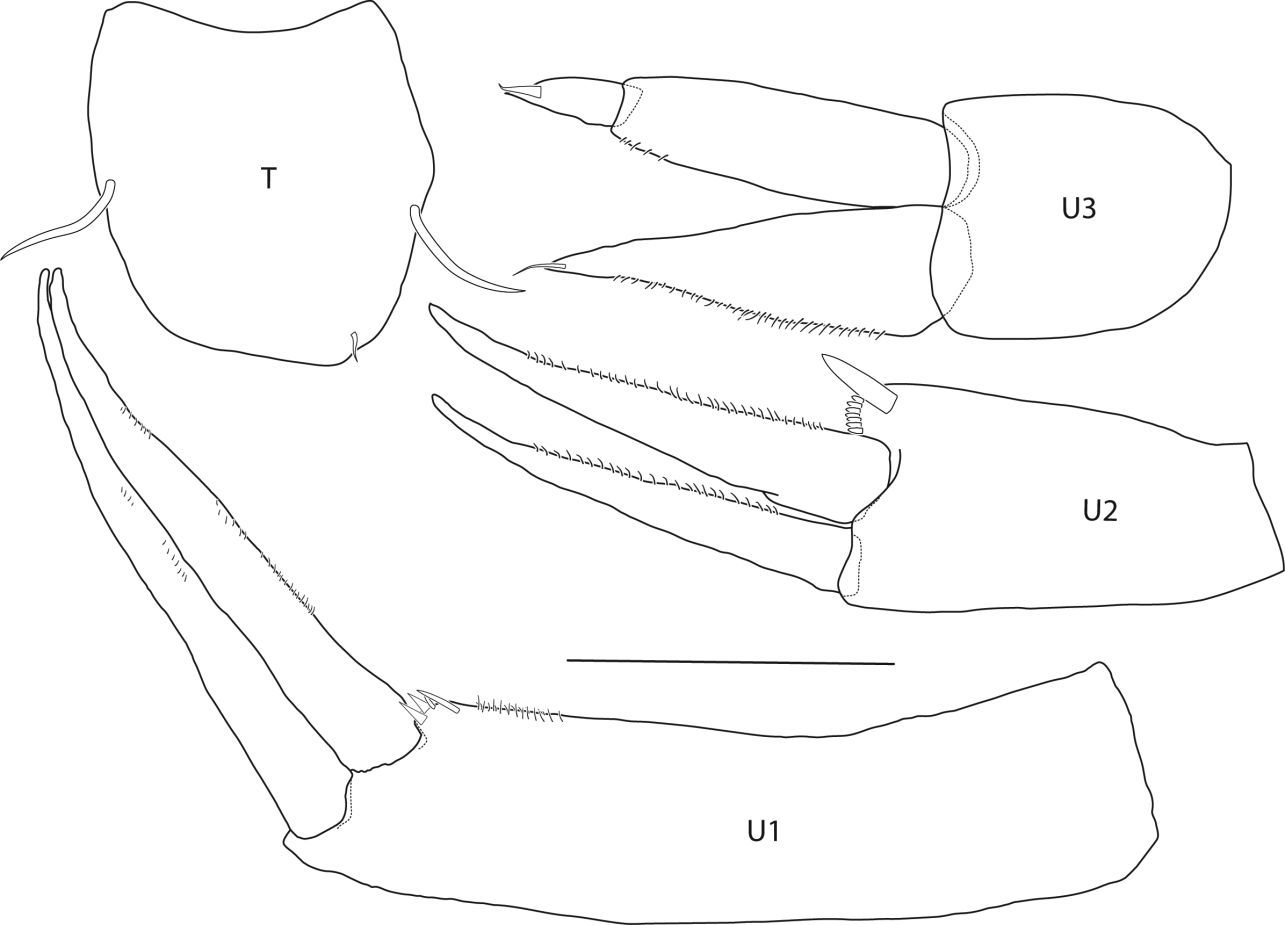
*Eperopeus vermiculatus* sp. nov., female holotype (with setose oostegites), 3.6 mm; NHMUK 2025.28. Scale bar: 0.1 mm (T, U1–U3).

***Urosome*: *Uropod 1*** (Fig. 5) peduncle length 1.36 × rami, dorsolateral corner with three or four small spines; rami lanceolate, equal in length, lacking spines. ***Uropod 2*** (Fig. 5) Peduncle subequal in length to outer ramus, dorsomedial margin with one strong spine and seven very small spines; rami lanceolate, inner ramus longer than peduncle and outer ramus, margins microsetose, both lacking spines. ***Uropod 3*** (Fig. 5) Peduncle short, length 0.65 × biarticulate outer ramus; outer ramus cylindrical, second article short, 0.26 × length of article 1; inner ramus slightly shorter than outer, strongly tapering with margin microsetose, with one apical seta. ***Telson*** (Fig. 5): Short, entire, length 1.1 × width, lateral margins with one submarginal seta, distal margin bluntly rounded, straight.

***Oostegites*** (Fig. 4): present on pereopods 3–5, short, subrectangular with two or three medium length apical brood setae.

***Gills*** (Fig. 4): present on pereopods 3–6. Subovate to cylindrical, shorter than basis.

**Male**: as for female except ***Antenna 1*** (Fig. 2): peduncular article 1 length 2.2 × width; callynophore present. Accessory flagellum with a single flattened, broad, scale-like article, distal articles not apparent.

##### Etymology.

The species name, *vermiculatus*, is from the Latin, meaning “like worms” or “of worms”. It was chosen to honour both the vermiform morphology of this new amphipod, and in recognition of the World Register of Marine Species (WoRMS 2025), a wonderful resource for all marine taxonomists.

##### Remarks.

This is the second species of *Eperopeus* described and the first record of this rarely reported genus from the Pacific Ocean. A list of differences between the two species is provided in Table 2. These differences are minor but allow for the separation of the two species (for which a key is also provided).

**Table 2.** Differences in morphological character state between *Eperopeus abyssicola* and *E. vermiculatus* sp. nov.

	***E. abyssicola***	***E. vermiculatus* sp. nov**.
**Mandibular palp**	Article 1 short, 0.5 × length of article 2	Article 1 elongate, 0.9 × length of article 2
**Maxilla 1**	Palp not expanded (erroneously shown as three-articulate)	Palp widened, two-articulate
**Gnathopod 2 carpus**	Broad, very similar to gnathopod 1	Narrower than that of gnathopod 1
**Gnathopod dactyls**	With strong teeth on inner margin	Teeth absent
**Pereopods 3, 4**	Dactyls equal to or longer than propodus	Dactyls shorter than (0.8–0.9 ×) propodus
**Pereopods 5, 6**	Dactyls very long, 2.2–2.3 × propodus	Dactyls 1.2 × propodus
**Uropod 1**	Rami length 0.6 × peduncle	Rami length 0.8 × peduncle
**Telson**	Length 0.75 × width	Length 1.1 × width

##### Molecular identification.

The species has received a Barcode Index Number from Barcode of Life Data Systems: BOLD:AEB4130 (https://doi.org/10.5883/BOLD:AEB4130).

##### Distribution.

Abyssal Pacific Ocean, Clarion-Clipperton Zone, 4132–4302 m.

### Key to the species of *Eperopeus*

**Table:** 

1	Gnathopod dactyls inner margin with strong teeth; pereopods 3–4 dactyls equal to or longer than respective propodus; pereopods 5–6 dactyls very long, > 2 × length of propodus; telson shorter than wide, truncate distally, length 0.75 × width; mandibular palp article 1 short, half-length of article 2	***Eperopeus abyssicola* Mills, 1967**
–	Gnathopod dactyls inner margin lacking teeth; pereopods 3–4 dactyls short, length 0.8–0.9 × than respective propodus; pereopods 5–6 dactyls length 1.2 × propodus; telson longer than wide, bluntly rounded distally, length 1.1 × width; mandibular palp article 1 elongated, 0.9 × length of article 2	***E. vermiculatus* sp. nov**.

## Supplementary Material

XML Treatment for Eperopeus

XML Treatment for Eperopeus vermiculatus

## Additional information

### Author ORCIDs

Tammy Horton https://orcid.org/0000-0003-4250-1068

Georgina Valls Domedel https://orcid.org/0009-0005-8932-6641

Eva C. D. Stewart https://orcid.org/0000-0001-8383-5705

Ed A. Hendrycks https://orcid.org/0000-0001-6199-7121

### Data availability

All of the data that support the findings of this study are available in the main text.
